# An overview of potential of natural compounds to regulate epigenetic modifications in colorectal cancer: a recent update

**DOI:** 10.1080/15592294.2025.2491316

**Published:** 2025-04-16

**Authors:** Susmita Roy, Dikshita Deka, Suresh Babu Kondaveeti, Pavithra Ayyadurai, Sravani Siripragada, Neha Philip, Surajit Pathak, Asim K. Duttaroy, Antara Banerjee

**Affiliations:** a Medical Biotechnology Lab, Faculty of Allied Health Sciences, Chettinad Academy of Research and Education (CARE), Chettinad Hospital and Research Institute (CHRI), Chennai, India; b Department of Biochemistry, Symbiosis Medical College for Women, Symbiosis International (Deemed University), Pune, India; c Department of Nutrition, Institute of Medical Sciences, Faculty of Medicine, University of Oslo, Oslo, Norway

**Keywords:** Phytochemicals, DNA methylation, histone modification, miRNAs, chromatin remodeling, cancer therapy, epigenetics

## Abstract

Colorectal cancer (CRC) remains an alarming global health concern despite advancements in treatment modalities over recent decades. Among the various factors contributing to CRC, this review emphasizes the critical role of epigenetic mechanisms in its pathogenesis and progression. This review also describes the potential role of natural compounds in altering the epigenetic landscape, focused mainly on DNA methylation, histone modification, and non-coding RNAs. Publications from the previous five years were searched and retrieved using well-known search engines and databases like PubMed, Google Scholar, and ScienceDirect. Keywords like CRC/colorectal cancer, CAC/Colitis associated CRC, inflammasomes, epigenetic modulation, genistein, curcumin, quercetin, resveratrol, anthocyanins, sulforaphane, and epigallocatechin-3-gallate were used in various combinations during the search. These natural compounds predominantly affect pathways such as Wnt/β-catenin, NF-κB, and PI3K/AKT to suppress CRC cell proliferation and oxidative stress and enhance anti-inflammation and apoptosis. However, their clinical use is restricted due to their low bioavailability. However, multiple methods exist to overcome challenges like this, including but not limited to structural modifications, nanoparticle encapsulations, bio-enhancers, and novel advanced delivery systems. These methods improve their potential as supportive therapies that target CRC progression epigenetically with fewer side effects. Current research focuses on enhancing epigenetic targeting to control CRC progression while minimizing side effects, emphasizing improved specificity, bioavailability, and efficacy as standalone or synergistic therapies.

## Introduction

Colorectal cancer (CRC) is the second most common cause of cancer-related deaths and is the third most commonly diagnosed cancer worldwide. CRC accounts for approximately 10% of all cancer occurrences globally and 9.4% of cancer-related mortality. It is estimated that by 2035, there will be more than double the number of CRC incidences, with 1.1 million deaths, especially in less developed countries. The rise in CRC cases and fatalities is driven by environmental factors, sedentary lifestyles, and dietary changes, along with genetic and epigenetic influences [1,2]. Lifestyle choices, the effectiveness of screening methods, and the accessibility of healthcare services largely influence the incidence and mortality rates of CRC. Mitigating the impact of CRC requires sustained efforts in public health education, early screening programs, and promoting lifestyle modifications. Appropriate screening and surveillance can reduce CRC mortality and morbidity [2]. Cancer cell formation results from multiple epigenetic and non-epigenetic modulations [3]. Epigenetic factors are modifications that alter gene expression without changing the DNA sequence [4,5]. Epigenetic events include DNA methylation, histone modifications, chromatin remodeling, and the regulatory effects of noncoding RNAs. Epigenetics, a reversible process, regulates both positive and negative traits. On the positive side, it governs processes like cellular growth and differentiation. On the negative side, it can also contribute to the development of certain diseases that may be inherited [6]. Despite being in similar stages and grades of the disease, CRC patients exhibit significant cellular heterogeneity that influences the pattern and progression of their condition. Epigenetic differences largely drive this heterogeneity at the cellular level, including various gene silencing mechanisms [7]. Sudden histone modifications can activate oncogenes and inhibit the tumor suppressor genes across various diseases, including CRC. Evidence from a study suggests that moderate to well-differentiated colonic cancer had heightened levels of acetylation of H3K18ac and H3K12ac, while poorly differentiated colonic cancer had lower levels. Their findings also indicated an elevated level of HDAC2 in adenocarcinoma compared to adenoma, highlighting its role in the transition from adenoma to adenocarcinoma [8]. A study demonstrated an increased methylation level of trimethylated lysine 9 on histone 3 (H3K9me3), particularly in invasive sites of CRC tissues. It was also observed that H3K9 trimethylation was favorably related to lymph node metastasis. Similarly, a study on a mouse model demonstrated that upregulated H3K9 methyltransferase SUV39H1 induced CRC development and resulted in a poor survival rate [8,9]. Gene promoter sites are rich in 5’-C-phosphate-G-3’ (CpG) dinucleotides and are commonly referred to as CpG islands. Methylation at the promoter region can regulate gene expression, and commonly, methylation occurs at the cytosine bases of CpG dinucleotides. Methylation dysregulation is often associated with aging, chronic inflammation, other stimuli etc. Evidence from the studies suggests that hypermethylation of promoter sites could result in the inhibition of TSGs and induce carcinogenesis of various cancers, including CRC [10]. Comparative research conducted between the normal and aberrant functions of cells due to epigenetic modulation can elucidate the potential treatment for various diseases, including cancer. Many cancer cells have aberrant genomic changes, such as genome instability, mutations, or epigenome dysregulation, which disrupt cellular function and promote tumorigenesis [11]. Therefore, comprehending the underlying mechanism of tumor development through epigenetics is crucial to enhancing the treatment strategies available for cancer patients. As a part of unconventional cancer treatment, natural compounds have greatly impacted drug development. Compounds derived from natural sources depict several beneficial roles in human health; for instance, a tetrahydroisoquinoline alkaloid ecteinascidin-743 (ET-743) derived from marine sources shows an anti-tumor effect [12]. Natural compounds are well-tolerated and exhibit lower toxicity, aiding patients in achieving better treatment outcomes and enhancing their quality of life. Natural compound-derived drugs have a wide range of applications in treating CRC [13]. An active effort is being made to restore the cellular memory lost during the tumor transformations, and pharmacological screening of natural compounds is being made against various epigenetic pathways, including histone methylation, DNA methylation, and chromatin remodeling, which has elevated due to the flexibility and plasticity of epigenetic modifications. These compounds inhibit DNMTs and alter histone and chromatin structures to re-establish the normal cell identity [14]. When combined with chemotherapeutic agents, natural compounds exert synergistic, preventive, and therapeutic effects. Therefore, these compounds could serve as supplementary therapeutic agents and be used as palliative therapy for various cancers and inflammatory diseases, where chemotherapy often causes many adverse effects when administered in high doses [15]. This narrative review has discussed the findings from various studies on the potential of targeting epigenetics in CRC therapy, focusing on epigenetic drugs (epi-drugs), small synthetic compounds, and epigenetic diets (epi-diets), natural compounds that help reverse the epigenetic instability associated with CRC carcinogenesis. Several dietary agents and plant polyphenols have demonstrated significant effects in slowing cancer progression, with some discussed here. Furthermore, it emphasizes the need to examine the molecular pathogenesis of CRC to identify additional targets for epigenetic therapy using natural products.

## Molecular pathogenesis of CRC

CRC is a malignancy that originates in the colon region of the large intestine, often involving the rectal tissue, and has the potential to invade other tissues through metastasis. The formation and advancement of colorectal adenomas and invasive adenocarcinomas are caused by the gradual accumulation of histological, molecular, genetic, and epigenetic changes in the normal colonic epithelium [16]. Numerous pathways contribute to the development of CRC, including microsatellite instability (MSI), chromosomal instability (CIN), and the CpG Island Methylator Phenotype (CIMP), each playing vital roles in genetic and epigenetic changes. Three key pathways have been identified that indicate the progression of CRC: chromosomal instability (which falls entirely within the non-hypermutated category), CIMP (associated with both hypermutated and non-hypermutated categories), and microsatellite instability (linked to the hypermutated category) [17,18]. Sequentially accumulating mutations in critical pathways such as Wnt, TGF-β, p53, and EGFR induces CRC initiation and progression. About 70% of colorectal adenomas have *APC* gene mutations, which can advance to carcinoma by activating mutations in *KRAS* and subsequent inactivation of TSGs like *Tp53* and *SMAD4* [19]. Multiple stages are involved in the development of CRC, initially starting with benign precancerous polyps in the inner lining of the large intestine and rectum and then gradually extending into the intestinal lumen. Then, it progresses into adenoma carcinoma *in situ* and eventually invasive carcinoma, characterized by distant metastasis in the final stage of development. Early detection of these polyps is crucial to interrupt the adenoma-carcinoma sequence and prevent the development of CRC [20].

Additionally, Colitis-associated colorectal cancer (CAC) is an inflammation-driven subtype of CRC and a significant complication in patients with ulcerative colitis (UC) and Crohn’s disease (CD). Chronic inflammation contributes to tissue damage and promotes tumor development [21]. Chronic inflammation is a key contributing factor in the development of CRC [22]. Inflammasomes are cytosolic multiprotein complexes that play a vital role in the inflammatory response and are essential for maintaining gut homeostasis [23]. Activated inflammasomes trigger caspase-1 activation, releasing effector cytokines like interleukin (IL)-1β and IL-18. However, their role in cancer is intricate and context-dependent, displaying both pro-tumorigenic and anti-tumorigenic effects [24]. During colitis and CAC, these cytokines are key in regulating the inflammatory response [23]. Multiple interconnected pathways simultaneously contribute to activating autophagy, inflammasomes, and epigenetic modifications in CRC [21]. Mutations and molecular changes in CAC are linked to inflammation driven by dysregulated inflammatory mediators and cytokines associated with IBD, leading to disruptions in cell communication, adhesion, and signalling [25]. Extensive evidence indicates that the (Nucleotide-binding domain, Leucine-Rich – containing family, Pyrin domain – containing-3) NLRP3 inflammasome plays a dual role in CRC, influencing tumor progression, prognosis, and treatment response while also contributing to tumor suppression by regulating host immunity. Overexpression of NLRP3 has been linked to poor prognosis and shorter survival in CRC patients. Additionally, genetic studies suggest that alterations in the NF-κB pathway affect CRC outcomes. Another study identified NLRP3 as a key factor in epithelial-mesenchymal transition, highlighting its role in enhancing cell migration and proliferation in CRC [24,25]. Understanding the molecular pathogenesis of CRC is essential for developing effective prevention, detection, and treatment strategies. Continued research into the genetic and epigenetic mechanisms underlying CRC will provide deeper insights into its progression and open up new avenues for therapeutic approaches.

## Epigenetic factors regulation in normal colon

The gut epithelium continues to be the ideal model for investigating the processes of normal and pathological differentiation processes. The single-layered mammalian intestinal epithelium comprises distinct villi and proliferating crypts. Proliferating and quiescent stem cells can self-renew and generate all differentiated cell types. These cells form the crypts, replenishing every 2 to 5 days [26]. The tumor suppressor-associated genes reduced expression due to hypermethylation at their promoter regions, leading to transcriptional silencing [27]. Genome-wide methylation analysis of miR genes in primary CRC and normal tissues identified 18 miR genes with differential methylation. Genes such as *miR124–2, miR129–2, miR124–3, miR137, miR34C, miR34B, miR548G, miR9–3* and *miR762* were hypermethylated, while *miR1204, miR17HG, miR17, miR18A, miR19A, miR19B1, miR548F5, miR20A, and miR548I4* were hypomethylated in CRC tumors compared to normal tissues. Most of these methylation changes in miR genes shown to contribute to CRC progression and metastasis [28]. One of the most important pathways is the Notch pathway, which regulates intestinal differentiation and proliferation. Notch signaling relies on contact between a ligand-presenting and a receptor-expressing cell, triggering the release of the Notch intracellular domain (NCID). NCID binds to the DNA-binding protein RBPJ, inducing expression of Hairy and enhancer of split 1 gene (*HES1*), suppressing TFs that promote differentiation, including the prosecretory agent like ATOH1. Intestinal stem cells require active Notch signaling, primarily provided by neighboring Paneth cells, to preserve their identity. Genetic (DLL1/DLL4 deletion) and chemical inhibition of Notch leads to stem cell loss through differentiation into secretory cells [29].

## Epigenetic modifications in CRC: causes and consequences

Numerous attributions are leading to CRC and its progression. In this review, we focus on one of the leading and highly influenced factors causing CRC, the epigenetic contribution. As [30] defines epigenetics as the additional modifications or changes that take place in the genome of an organism without the original DNA nucleotide sequence itself being altered. These changes influence the daughter cells that are being produced [30]. The genetic and epigenetic instabilities promote CRC development. The chromosomal instability (CIN) is one of the main drivers of CRC, accounting for about 80%-90% of CRC cases, whereas microsatellite instability (MSI) accounts for approximately 15%-20%. The findings from studies suggest that about 20% of MSI cases are hereditary due to germline mutations in mismatch repair genes, such as *PMS2, MLH1, MSH2*, and *MSH6*.

**Figure 1.**
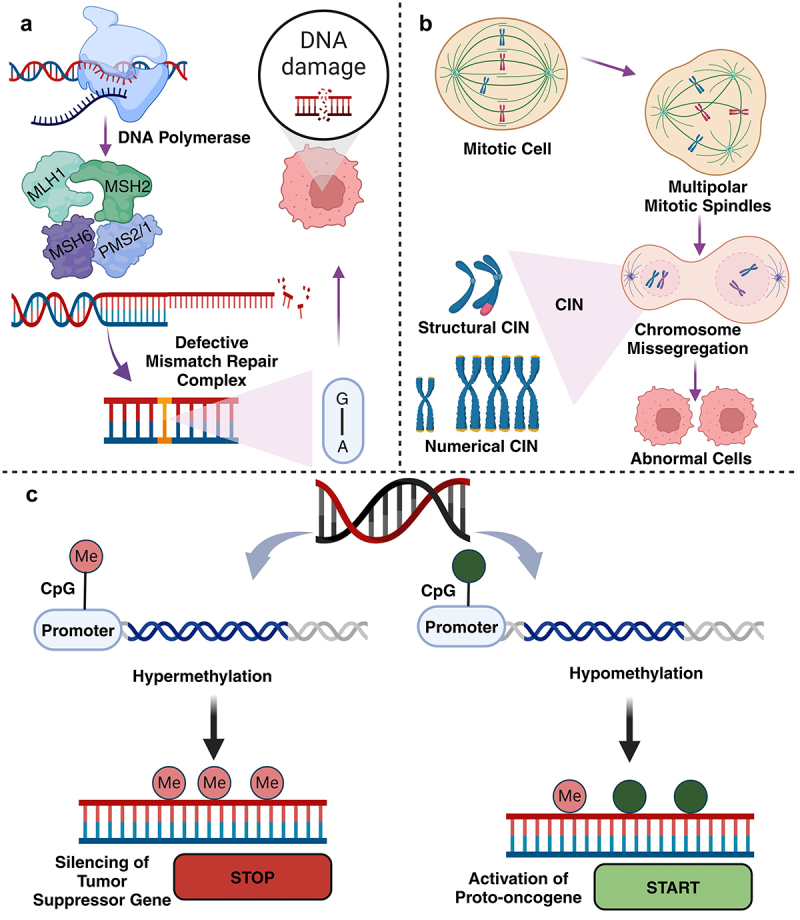
Illustrates the (a) microsatellite instability as a result of defective DNA mismatch repair process, (b) role of chromosomal instability (CIN) in CRC progression, (c) epigenetic instability involves aberrant methylation of tumor suppressor genes. Figure created using BioRender.com.

Additionally, it was observed that hypermethylation of promoter regions of TSGs leads to epigenetic silencing and contributes to approximately 20%–30% of CRC cases [31]. The 5’ region of the gene with repeated CpG dinucleotides, known as CpG islands, are highly methylated at the time of cancer pathogenesis. Along with methylation, these regions are also very susceptible to transcriptional repression as depicted in (Figure 1). A subtype of CRC, referred to as the CpG island methylator phenotype (CIMP), is characterized by widespread gene methylation and a distinct epigenetic profile. Prior studies have noted that upregulation of oncogenes and proto-oncogenes due to hypermethylation of CpG island promoters is more common than hypomethylation of non-CpG island promoters [32]. The risk of metastasis in CRC is heightened by a condition known as a lymphovascular invasion (LVI), defined by the presence of cancer cells within the lymphatic and blood vessels of the primary tumor. This condition indicates the tumor’s capacity to intravasate, a critical step in metastasis. In CRC patients with lymph node metastasis or LVI, tumor suppressor genes (TSGs) such as *FAM134B* and *CDKN2A* have been found to be hypermethylated [33]. Hypermethylation of CpG islands in the promoter regions is more prevalent in tumor samples than in normal samples, leading to altered gene expression of transcription factors (TFs) and their target genes, contributing to colon cancer progression. A study has observed that TFs are significantly enriched. These points to an underlying cellular mechanism by which tumor cells change the expression of numerous genes by methylating only a few. Activation of TFs in tumors due to promoter hypomethylation generally relates to the development of transcriptional regulation, including *PITX1, PITX2, MSX1, MSX2* and *SIM2*. *PITX2* upregulation has been observed in various cancer types. In an *in vivo* colon cancer model, the knockdown of *PITX2* demonstrated an inverse correlation between its expression levels and the growth and invasion of colon cancer cells [34].

## Currently available treatments for CRC

The selection of treatment method is based on many underlying issues associated with tumor progression, metastases, biochemical markers, prognosis, and staging of CRC. Surgery is opted for patients with no visible metastasis. Patients with potentially removable metastases are treated with chemotherapy to reduce the size and the progression of metastases and then subjected to successive surgical procedures. In CRC treatment, some of the drugs aim to inhibit VEGF function. Vascular endothelial growth factor (VEGF) is an angiogenic growth factor that supports endothelial cell survival, migration, and proliferation. A synergistic effect is observed when combining anti-VEGF and anti-EGFR therapies, as these drugs target common downstream signaling pathways. Cetuximab and VEGF antisense oligonucleotides work synergistically to inhibit EGFR and VEGF efficiently, increasing anticancer efficacy and boosting survival in mice with human GEO colon cancer cell xenografts [35]. In a clinical trial AVF2107g, the monoclonal antibody bevacizumab, combined with fluorouracil-based chemotherapy, effectively blocks VEGF in treating CRC. Patients in the trial were administered bolus fluorouracil, irinotecan, and leucovorin (IFL), plus a placebo, plus bevacizumab or an IFL, as a first-line therapy. It has been found that the group that received IFL plus bevacizumab showed better outcomes when compared to a group that received IFL along with a placebo [36]. The standard chemotherapy agents used in CRC treatment include 5-FU, oxaliplatin, and irinotecan. However, a significant percentage of patients with colorectal cancer do not respond to these regimens and frequently suffer serious adverse effects. Several studies have shown that high microsatellite instability (MSI-H) results due to defective DNA mismatch repair processes, reducing the efficacy of 5-FU-based treatments compared to CRC patients with microsatellite stability [37]. EGFR, a member of the ErbB family of receptor tyrosine kinases, plays a key role in cellular processes like proliferation, migration, angiogenesis, survival, and adhesion. Due to its critical functions in cancer cell growth, EGFR is an important therapeutic target for metastatic CRC. The FDA has approved cetuximab, an anti-EGFR monoclonal antibody, for EGFR-positive, *KRAS* wild-type metastatic CRC in combination with FOLFIRI as a first-line treatment. It is also combined with irinotecan for patients resistant to irinotecan-based chemotherapy. In cases where patients are intolerant to irinotecan or have failed oxaliplatin and irinotecan regimens, cetuximab is administered. The BOND study demonstrated cetuximab’s effectiveness against metastatic CRC, highlighting the targeting of the EGFR pathway as a crucial mechanism for overcoming chemotherapy resistance. The CRYSTAL trial further confirmed the efficacy of cetuximab plus FOLFIRI as the first-line treatment for EGFR-positive metastatic CRC [36]. In microsatellite, stable colorectal cancer (MSS-CRC), inhibition of DNMTs increases the expression of neoantigen-bearing genes, which promotes the increased presentation of neoantigens by MHC class I molecules on tumor cells. This, in turn, leads to heightened activation of neoantigen-specific T-cells when combined with radiotherapy [38]. DNA methylation inhibitors are classified into nucleoside analogs, non-nucleoside analogs, and anti-sense oligonucleotides (ASO). Modified cytidine nucleoside analogs, including 5-aza-2-deoxycytidine, 5-aza-cytidine, 5-fluoro-2-deoxycytidine, and zebularine, block DNMT enzymatic activity by forming covalent bonds with DNMTs upon incorporation into DNA [39]. Total neoadjuvant therapy (TNT), which combines chemotherapy and chemoradiotherapy before surgery, has been shown to improve rectal cancer outcomes. TNT significantly reduces distant metastases, enhances disease-free survival by 5–10% over three years, and increases overall survival by approximately 5% over seven years. Compared to standard neoadjuvant therapy, TNT achieves better results due to the combined chemotherapy approach, which in the trials included 6 cycles of CAPOX or 6–9 cycles of FOLFOX/FOLFOXIRI [40]. Treatment for metastatic CRC is made patient-specific, tailored to target predictive biomarkers, including *RAS* (*KRAS/NRAS*) and *BRAF* V600E mutations and a DNA mismatch repair status. The overall survival rate has significantly improved due to advancements in developing effective CRC drugs, allied specialties, and surgical procedures [41]. Despite significant progress in CRC treatment, chemotherapy and radiotherapy remain the primary treatment options. However, these mainstream regimens are associated with severe side effects, including nephrotoxicity, cardiotoxicity, neurotoxicity, gastrointestinal toxicity, hepatotoxicity, mucositis, myelosuppression, and alopecia, which can negatively impact the quality of life of cancer patients [42]. Compounds derived from plants and animals offer notable health benefits, including anticancer potential. Many of these compounds, particularly in preclinical studies, have been investigated in combination to assess their effectiveness in cancer prevention and treatment. These combinations include plant-based compounds such as polyphenols, phytosterols, triterpenoids, and saponins; animal-derived compounds like vitamin D and omega-3 fatty acids; and compounds sourced from both plants and animals, such as carotenoids. The subsequent sections outline a selection of natural compounds exhibiting single-agent efficacy. The list is not intended to be exhaustive. Natural compounds from plants and animals, such as plant-derived polyphenols, phytosterols, triterpenoids, saponins, and animal-derived vitamin D, omega-3 fatty acids, and carotenoids, have shown immense health benefits. Additionally, melatonin has shown potential in cancer treatment. Combination therapies are increasingly recognized for their greater effectiveness compared to single-agent therapies, with the primary aim of maximizing efficacy while minimizing side effects. Research now focuses on developing agents from natural compounds, with notable success already attained [43]. In addition to traditional treatment methods, alternative therapies are being examined and studied to enhance treatment effectiveness and reduce adverse effects. A correlation has been detected in the utilization of anti-inflammatory medications, tumor macro beads, probiotics, metal-based medications, and medications derived from natural sources. A significant advancement in recent years has been identifying the potential to specifically target epigenetic enzymes and other elements involved in epigenetic regulation to treat CRC [44,45]. Some drugs targeting epigenetic regulators in CRC are listed in Table 1.

**Table 1.** Drugs targeting epigenetic regulators in CRC.

Name of the drug	Targets epigenetic regulators	Possible mechanism of action	Reference
Vorinostat or suberoylanilide hydroxamic acid (SAHA)	Inhibits HDAC class I and II	ROS dependent apoptosis, suppresses (HIF)-1 alpha and VEGF and hence blocks angiogenesis. Targeting HDAC in CRC treatment	[46]
Domatinostat (4SC-202)	Inhibits HDACs HDAC1, HDAC2 and HDAC3. Also inhibits lysine demethylase (KDM)	Targets Hedgehog (HH)/Gli signaling pathway, inhibits TGF-beta induced epithelial-to-mesenchymal transition (EMT), and induces TSG *p21* expression	[46]
Resminostat (4SC-201) (in Clinical Trials)	Inhibits HDAC classes I and II including HDACs 1, 3, 6, and 8	Targets AKT signaling pathway	[46]
Belinostat	Inhibits HDAC	Silences TGF-beta and targets PKA pathway	[46]
Panobinostat (LBH589)	Inhibits HDAC including classes I, II and IV	In CRC, Panobinostat activates death-associated protein kinase (DAPK) to induce autophagy and apoptosis and downregulates EGFR, HER2, and HER3 at mRNA and protein levels.	[46]
Zebularine [1-beta-D-ribofuranosyl-2(1H)-pyrimidinone]	Inhibits DNMTs	Inhibit DNA methylation. Induces p53 dependent ER stress and autophagy, inhibits tumor growth and stemness and increases let-7b a tumor suppressor miRNA to reduce cell invasion.	[46]
Disulfiram (DSF, bis-diethylthiocarbamoyl disulfide) or Antabuse	Inhibits DNMTs	DSF combined with 5-FU enhances chemosensitivity, apoptosis, and 5-FU toxicity. DSF also inhibits NF-кB nuclear translocation and DNA binding, countering chemoresistance in cancer cells.	[46]
Decitabine (5-aza-2’-deoxycytidine)	Inhibits DNMTs	Inhibits CRC by upregulating NALP1 expression. Reduces invasion in SW620 cells, promotes epithelial traits, and works synergistically with gefitinib and azacitidine in treatment of CRC.	[46]
Azacitidine or Vidaza	Inhibits DNMTs	Targets MDA5/MAVS/IRF7 pathway. Hypomethylates the NDN promoter, increasing NDN expression, binds to LRP6, reduces transcription and inhibits Wnt signaling in CRC.	[46]
Chaetocin	Inhibits HMTs	Induces ROS accumulation and activates c-Jun N-terminal kinase (JNK)/c-Jun pathway in CRC.	[47]

## Drugs targeting epigenetic regulation of cancer cells

Epigenetics refers to heritable changes to the genome that occur without alterations to the DNA sequence. Epi-drugs are small-molecule inhibitors that target epigenetic modifying enzymes, inducing programmed tumor cell death by affecting processes such as the cell cycle, proliferation, angiogenesis, and migration. Epi-drugs are currently the subject of extensive research, development, and application [48]. Epi-drugs, including DNA methyltransferase inhibitors (DNMTIs), agents targeting noncoding RNAs like microRNAs, chromatin remodelers, and HDAC inhibitors, are key therapeutic agents for cancer treatment. Approved by the FDA, these drugs are undergoing further trials to improve efficacy. When combined with standard therapies such as chemotherapy, epi-drugs have shown promising outcomes. Research into biomarkers for epi-drug sensitivity offers promising avenues for identifying responsive patients. While epi-drugs demonstrate strong anti-tumor effects, patient sensitivity remains a challenge. Notably, the combination of epi-drugs with chemotherapy has enhanced anti-tumoral effects, overcome drug resistance, and activated immune responses [49].

### Mechanism of epi-drugs

Epigenetic modifications, including DNA methylation, histone acetylation, and histone methylation, regulate gene expression at the transcriptional level by upregulating, downregulating, or completely silencing genes. Pathological dysregulation of these epigenetic processes contributes to the development of diseases such as cancer, metabolic disorders, neurological conditions, and cardiovascular diseases [50]. Epi-drugs primarily function by targeting enzymes that regulate epigenetic modifications to preserve normal cellular function. These include DNA methyltransferase inhibitors and histone deacetylase inhibitors, among others. Epi-drugs can potentially enhance treatment effectiveness and sensitize cancer cells resistant to conventional therapies, including chemotherapy, radiotherapy, and immunotherapy by working synergistically [51]. Epi-drugs primarily function by inhibiting DNMTs and HDACs, enzymes essential for establishing and maintaining epigenetic modifications [52]. Cancer is a complex, multifactorial disease marked by molecular evolution. It has been indicated that miRNA expression profiles and their dysregulation are linked to specific malignancies. These modifications can either inhibit or promote the expression of various cancer-related genes. Consequently, miRNAs are considered potential tumor markers for both diagnostic and therapeutic applications [53]. Tumorigenesis is a heterogeneous process characterized by genetic alterations and epigenetic silencing of miRNA genes through mechanisms such as DNA promoter histone hyperacetylation or hypomethylation, observed in various cancer types [54]. Moreover, miRNA expression profiling is crucial for classifying prognosis and treatment responses. Numerous pharmaceutical companies are actively engaged in identifying and discovering various miRNAs and their mimics associated with multiple cancer types, particularly CRC [55]. The U.S. Food and Drug Administration (FDA) has approved some epi-drugs that are used to treat cancer. However, new potential epi-drug candidates are continuously assessed for their cytotoxicity, pharmacological properties, and mechanisms of action through preclinical research *in vitro* and *in vivo* and clinical trials, aiming to facilitate the development and approval of novel therapies.

### Limitations of Epi-drugs and conventional CRC treatment regimens

Epi-drugs, targeting reversible DNA and histone modifications to regulate gene expression, hold promising therapeutic potential, but their clinical application faces several key challenges. One major issue is specificity, as epi-drugs can impact multiple genes and pathways, resulting in off-target effects and potential therapy resistance [51,56]. Clinical trials with first-generation epi-drugs have shown limited success, with only a few advancing to regulatory approval. For example, FDA-approved DNMT inhibitors azacytidine and decitabine have shown only 16–17% efficiency, possibly due to their global demethylation activity, which upregulates not only TSGs but also oncogenes. Identifying relevant biomarkers is crucial for achieving more targeted epi-drug therapies. Furthermore, concerns about the long-term effects of altering epigenetic marks remain, as do challenges in identifying suitable surrogate tissues for studying epigenetic changes in CRC [54]. Current chemotherapy regimens for CRC include both single-agent and combination therapies.

**Figure 2.**
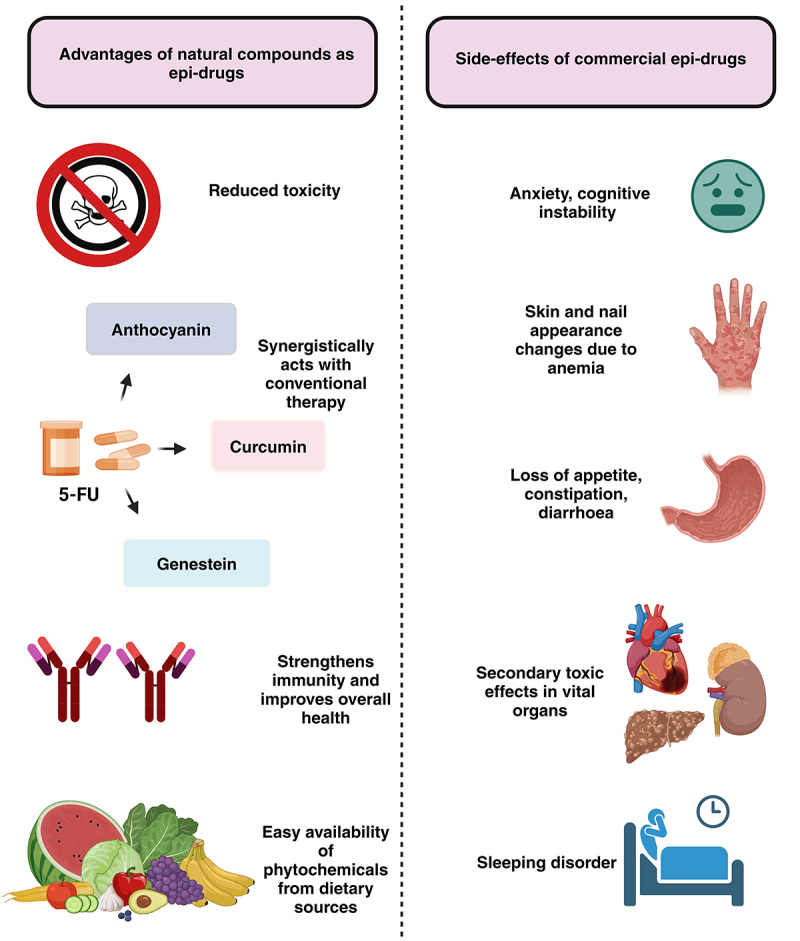
Depicts the advantages of natural compounds as epi-drugs and side-effects of commercially available epi-drugs regulating CRC. Figure created using BioRender.com.

Single-agent therapy typically involves 5-FU, while combination therapies use drugs like irinotecan (IRI), oxaliplatin (OX), and capecitabine (CAP or XELODA or XEL). However, studies have debated that single-agent therapy can offer similar overall survival rates to combination treatments in terms of overall survival. The first-line combinations such as FOLFIRI (5-FU + IRI), FOLFOX (5-FU + OX), and XELOX or CAPOX (CAP + OX), CAPIRI (CAP + IRI), remain the primary treatment approach. Patients with poor performance or lower risk of disease progression may be advised to receive single-agent therapy. Chemotherapy has extended the overall survival of metastatic CRC patients to about 20 months, solidifying its role as the treatment foundation. However, limitations such as systemic toxicity, low tumor selectivity, response variability, and resistance highlight the need for new therapeutic approaches [40,57]. The limitations of both epi-drugs and conventional chemotherapy highlight the need for novel, targeted treatments with improved safety and efficacy. Although many epi-drugs have been developed for CRC treatment, their long-term side effects remain unclear. The field of research is still in its infancy, and further study is needed to obtain a more comprehensive understanding. The lack of reliable biomarkers for patient stratification and treatment monitoring further restricts their clinical application. In light of these challenges, natural compounds present a compelling alternative characterized by their potential for reduced toxicity and increased specificity. As research in this area progresses, natural compounds may play a vital role in the future of cancer therapy, addressing current therapeutic gaps and enhancing treatment outcomes. Natural compounds also hold the potential to overcome these limitations and harmful side effects while acting through similar epigenetic mechanisms, as illustrated in (Figure 2).

## Natural compounds targeting specific epigenetic factors

There have been many other dietary compounds and phytochemicals which were observed to distinctively target epigenetic modifications in CRC, some of which have been mentioned below.

### Targeting Histones

Glycerol trihexanoate (TCN), or tricaproin, is derived from the chloroform extract of *Simarouba glauca* leaves and has shown anticancer effects in CRC models. TCN induces apoptosis by decreasing the activity of the oncogenic enzyme HDAC1 in HCT-116 and HCT-15 cancer cells [58]. A study has highlighted that the diallyl disulfide, an organosulfur compound in garlic, induces histone H4 and/or histone H3 acetylation in the CDKN1A promoter region and results in elevated expression of *CDKN1A* mRNA and *p21WAF1* in Caco-1 and HT-29 cancer cell lines, thus preventing cell proliferation and also causing cell cycle arrest [59]. A study on butyrate, a short-chain fatty acid produced by gut microbial fermentation of dietary fiber, found that it influences cellular mechanisms in a concentration-dependent manner. At low concentrations (0.5 mm), it serves as an energy source without affecting the HDAC activity. However, at higher concentrations (5 mm), it functions as an HDAC inhibitor [60]. 3,3’-diindolylmethane (DIM), a metabolite of indole-3-carbinol, abundantly found in broccoli or cauliflower, downregulated class 1 hDACs, resulting in cell cycle arrest and apoptosis. It has been highlighted in the study that DIM works synergistically with 5-FU and boosts its cell inhibitory effect in both *in vitro* and *in vivo* CRC models [61]. Kaempferol, a natural compound, reduces A4CT2 methylation by suppressing DNMT expression in HT-29 and HCT116 cell lines, facilitating gene transcription. It also downregulates key components of the Wnt signaling pathway, which functions downstream of A4CT2 and β-catenin in HCT116 cells. This leads to G1 phase cell cycle arrest and apoptosis induction [62]. Research on the benzoic acid derivative dihydroxybenzoic acid (DHBA) has demonstrated its ability to suppress HDAC expression, generate ROS, and inhibit the proliferation of colorectal cancer cell lines, including HCT115 and HCT116, in both *ex vivo* and *in vitro* settings [58].

These findings highlight the potential of natural compounds in targeting histone modifications to regulate CRC progression. Glycerol trihexanoate (TCN) induces apoptosis by inhibiting HDAC1, while diallyl disulfide and butyrate promote histone acetylation, leading to cell cycle arrest. Additionally, DIM enhances the efficacy of 5-FU by downregulating class 1 hDACs. Kaempferol and DHBA further contribute to epigenetic modulation by suppressing DNMT and HDAC expression, respectively, thereby inhibiting CRC cell proliferation and inducing apoptosis.

### DNA methylation

Cucurbitacin B, a triterpenoid isolated from the Cucurbitaceae family of plants, induced DNA demethylation in the promoter region of a TSG, B-cell translocation gene 3 (*BTG3*), thereby increasing its expression, resulting in the inhibition of cancer cell proliferation and stimulating cell apoptosis. It was also observed to reduce the levels of DNA methyltransferases (DNMTs) such as DNMT1, DNMT3a, and DNMT3b in both Caco-2 and SW480 cell lines, and the result was comparable to those of Aza-dC [63]. The effect of curcumin on DNA methylation in colon cancer cell lines, including HCT116, HT29, and RKO were evaluated by researchers. In contrast to 5-aza-CdR’s global hypomethylation, curcumin selectively demethylated specific CpG sites, providing new insights on its chemopreventive properties. Similar results from other research have also been published, showing that curcumin can epigenetically restore the expression of TSGs such *p21, DLEC1* and *RARβ* [64]. A study on kaempferol in CRC revealed its inhibitory effect on DNMTs, specifically DNMT1 and DNMT3B, while showing no impact on DNMT3A [65]. Recent research has provided deeper insights into the impact of natural compounds on epigenetic regulation in CRC. Sulforaphane, a phytochemical derived from cruciferous vegetables, inhibits DNMTs in Caco-2 cells, resulting in the demethylation of the Nrf2 promoter and increased Nrf2 expression. This elevation strengthens cellular defense against oxidative stress and helps suppress tumor development [66].

Overall, these findings highlight the potential of natural compounds in modulating DNA methylation to restore tumor suppressor gene expression and inhibit CRC progression. Cucurbitacin B induces DNA demethylation of the BTG3 promoter, restoring its expression and inhibiting CRC cell proliferation by downregulating DNMT1, DNMT3a, and DNMT3b. Curcumin selectively demethylates specific CpG sites, reactivating tumor suppressor genes like *p21, DLEC1*, and *RARβ*, unlike 5-aza-CdR’s global hypomethylation. Kaempferol inhibits DNMT1 and DNMT3B but has no effect on DNMT3A. Sulforaphane demethylates the Nrf2 promoter in Caco-2 cells, enhancing antioxidant defense and tumor suppression.

### Targeting microRNAs

**Figure 3.**
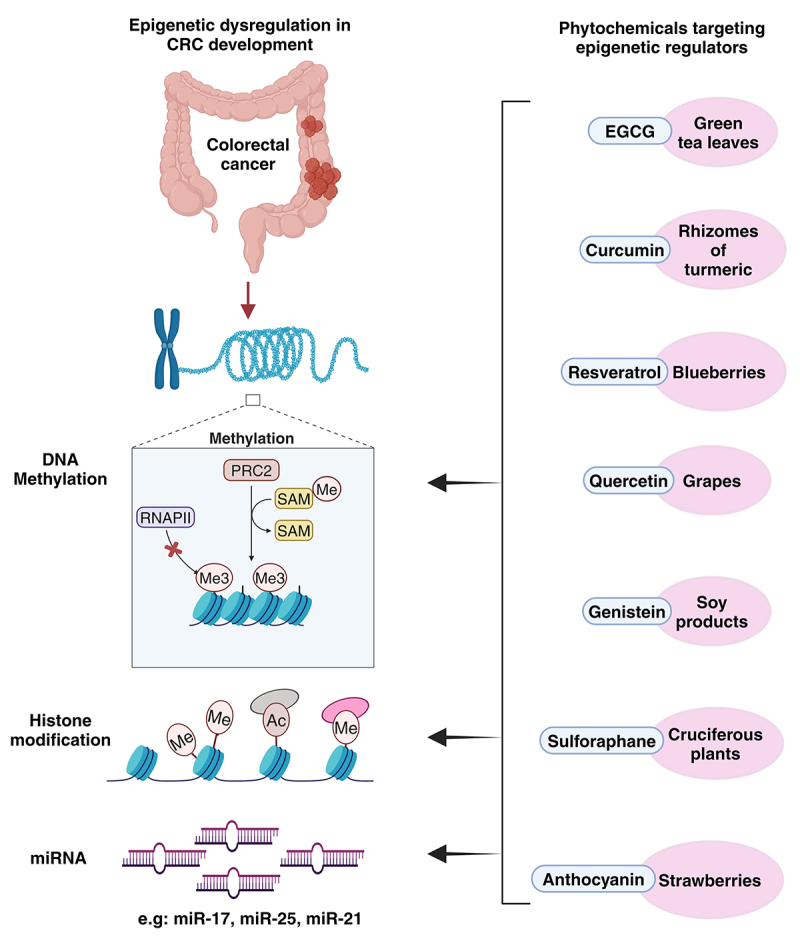
Role of natural compounds in targeting epigenetic modulators in CRC. Figure created using BioRender.com.

α-Mangostin, a xanthone derived from the pericarp of mangosteen fruit, induced apoptosis and anti-tumor effects in human CRC cells by increasing the expression level of miR-143, which regulates negatively regulates MAPK and Erk5 pathways [67]. Similarly, grape seed extract (GSE), a key polyphenol in red wine, has been linked to preventive effects against various cancers, including breast, prostate, skin, head and neck, lung, and colon, which might be due to its antioxidant and anti-inflammatory properties. A miRNA array revealed that long-term GSE supplementation in mice with colonic tumors led to the upregulation of miR-19a, let-7a, and miR-20a, and the downregulation of miR-103, miR-148a, miR-135b, miR-205, and miR-196a in the mouse colonic mucosa [67,68]. A study has shown that vitamin D (1,25(OH)_2_D_3_) regulates miR-22 in colon cancer cells in a time and dose-dependent manner, which adds to its anti-tumor, anti-proliferative and anti-migratory effects against colon cancer [69]. Another study reported that vitamin D led to the induction of miR-627, which inhibited the activity of the cytochrome P450 enzyme CYP3A4 in colorectal adenocarcinoma cell lines HT-29 and HCT-116. This effect was achieved by suppressing the histone demethylase JMJD1A, thereby contributing to anti-cancer activity by prolonging the retention of anti-cancer drugs [70]. Phytochemicals targeting the epigenetic dysregulation in CRC are illustrated in (Figure 3). A study reported that curcumin, a polyphenol extracted from *Curcuma longa*, has been found to downregulate the expression of miR-17-5p, miR-20a, and miR-27a in CRC cells, miRNAs implicated in oncogenic pathways. This regulatory effect highlights curcumin’s potential to interfere with tumor-promoting mechanisms, contributing to its anti-cancer properties [68]. Resveratrol regulates miRNA expression in colorectal cancer by upregulating the levels of miR-96, miR-101b, miR-455, miR-663, and miR-34a, which contribute to the downregulation of oncogenes and pro-inflammatory cytokines. This modulation inhibits tumor progression and facilitates apoptotic pathways [67,68]. Furthermore, oxyresveratrol, a natural resveratrol derivative, suppresses human colon cancer cell migration by modulating EMT and miRNA expression. In TGF-β-induced HT-29 cells, it downregulates miR-3687 and miR-301a-3p while upregulating miR-3612, ultimately inhibiting cell migration [71].

In summary, natural compounds modulate microRNAs to suppress CRC progression. α-Mangostin increases miR-143, inhibiting MAPK and Erk5 pathways, while GSE regulates multiple miRNAs linked to cancer prevention. Vitamin D regulates miR-22 in a dose-dependent manner and induces miR-627, enhancing anti-tumor effects and drug retention. Curcumin downregulates oncogenic miRNAs, resveratrol upregulates tumor-suppressive miRNAs, and oxyresveratrol modulates EMT-related miRNAs to inhibit cancer cell migration.

## Natural compounds as epigenetic regulators of CRC

Ethnopharmacology involves studying how various cultures and indigenous peoples use natural substances for medicinal purposes. Historically, ethnopharmacology was the foundation of all medicines and plant-based products, with knowledge passed down over generations. Driven by human curiosity, plant-derived natural products are now extensively utilized in therapies for treating various disorders. For centuries, these products have been valued as sources of therapeutic agents and structural diversity [72]. Identifying new anticancer agents from natural sources has become a central focus in pharmaceutical research, driven by the chemical diversity in plants, animals, and microorganisms. Over 60% of modern anticancer drugs have originated from natural sources. Plants and microbes are selected based on their phytochemical composition, ecology, and ethnopharmacological properties, with plants playing a significant role in developing effective anticancer treatments [73]. The use of plants, their parts, and their components, which have health benefits, have been preferentially used since ancient times. It has been shown that the intake of fruits and vegetables daily reduces the risk of an individual developing cancer. The accuracy and precision of components and their mechanisms must also be determined before the clinical trials.

### Genistein

Among many dietary components, soy phytoestrogen-originated isoflavones have been reported to show effects on tumorigenesis through many mechanisms. Genistein (C_15_H_10_O_5_) was identified as a primary bioactive compound in soy products having anti-cancer effects [74,75]. It has been indicated in a study that genistein could induce apoptosis in HT-29 and LoVo human colon cancer by upregulating Bax and downregulating the NF-κB pathway, thus supporting the probable application of genistein in clinical application for treating colon cancer patients [76]. Genistein demonstrates a range of biological activities, including anticancer properties, restraint of tyrosine kinase activity, and antioxidant activity. The anticancer property of genistein influences several cellular processes, including apoptosis, angiogenesis, and regulation of the cell cycle. Effects such as inducing apoptosis, blocking metastasis, and angiogenesis are exerted through epigenetic modifications targeting cancer-related genes, including lncRNAs [77]. Another study has highlighted the role of genistein in reducing the expression of the EED in PRC2, further disrupting the interaction between HOX transcript antisense RNA (HOTAIR) lncRNA and PRC2. This interference prevents the recruitment of the HOTAIR/PRC2 complex to the ZO-1 promoter, leading to increased transcription of the *ZO-1* tumor suppressor gene and potentially reducing cancer cell metastasis [74]. Genistein modulates DNA methylation and histone acetylation of genes across various cancer cell types. A study demonstrated that treatment with genistein in SW1116 colon cancer cells reactivated the Wnt5a gene, which acts as an antagonist to the Wnt signaling pathway, frequently overactivated in colon cancer.

Additionally, genistein has been shown to suppress the growth of colon cancer by suppressing miR-95, a microRNA that promotes tumorigenicity [78]. LncRNAs are commonly implicated in regulating cancer initiation, differentiation, proliferation, invasiveness, and metastasis. Targeting TTTY18 May offer a promising approach for managing metastatic CRC. A study demonstrated that treatment of SW480 cells with genistein promoted apoptosis, suppressed cell viability, reduced cellular migration, decreased Ki-67 positive cells, and downregulated the expressions of TTTY18 lncRNA, SGK1, Akt^Ser473^, and p38 MAPK^Tyr323^ [79]. Studies have shown that genistein enhances the expression of numerous Wnt antagonists, inhibiting the Wnt/β-catenin signaling pathway. It has also demonstrated that genistein treatment reduces CpG island methylation in the *WNT5A* and *SFRP2* genes, preventing the translocation of β-catenin into the nucleus. A study showed that genistein reduces histone H3 acetylation at the promoters of Wnt pathway antagonists (*Sfrp2, Sfrp5*, and *Wnt5a*) in colon tissues, reducing RNA polymerase II binding and gene expression. Additionally, it reduces H3K9Me3 and histone H3 serine 10 phosphorylation (H3S10P), highlighting its role in chromatin remodeling and gene regulation [80]. Another study on genistein showed that it inhibits CpG island methylation in the Wnt5a promoter region in colon cancer cell lines (DLD-1, SW480, and SW1116), resulting in elevated Wnt5a expression. Additionally, it downregulates the oncogenic miRNA miR-1260b, which directly targets sFRP2 in colon cancer cells [81]. Furthermore, another mechanism of Wnt pathway inhibition involves the increased acetylation of the promoter region of DKK1, a known Wnt pathway antagonist by histone H3 inhibition [82]. FDA classifies genistein as a generally recognized safe (GRAS) substance [83].

In brief, genistein, a soy-derived isoflavone, exhibits anticancer effects in CRC by modulating multiple epigenetic and signaling pathways. It induces apoptosis by upregulating Bax and inhibiting NF-κB while targeting lncRNAs and disrupting PRC2-HOTAIR interactions to restore tumor suppressor gene expression. Genistein also reactivates Wnt antagonists by inhibiting CpG island methylation and histone modifications, suppressing the Wnt/β-catenin pathway. Additionally, it downregulates oncogenic miRNAs, such as miR-95 and miR-1260b, further contributing to its anti-tumor properties. Recognized as safe by the FDA, genistein holds promise as a potential therapeutic agent for CRC.

### Curcumin

Curcumin, a polyphenolic compound from turmeric (*Curcuma longa*), inhibits DNMT activity in various cancer cell lines, including CRC. It modulates multiple signaling pathways, such as those involved in cell survival (Bcl-xL, Bcl-2, cFLIP, c-IAP1, XIAP), cell proliferation (cyclin D1, c-myc), tumor suppression (p53, p21), death receptor signaling (DR4, DR5), caspase activation (caspase-8, 3, 9), mitochondrial function, and protein kinase pathways (AKT, JNK, AMPK) [84]. Previous studies have identified curcumin as an epigenetic modulator. Specifically, curcumin treatment in HCT116 cells upregulates miR-491 and inhibits the PEG10 gene and also silences the Wnt/β-catenin signaling pathway [85]. Recently, miR-27a has been identified as a key oncogenic factor in CRC. Studies show that curcumin treatment increases miR-34a levels while reducing miR-27a expression and, as a result, suppresses CRC. Curcumin may reverse cancer cell resistance by modulating non-coding RNAs. It has also been demonstrated that curcumin enhances the effects of cisplatin by regulating miR-137, which inhibits glutamine metabolism in CRC. Additionally, curcumin helps overcome CRC resistance to oxaliplatin by influencing the miR-409-3p-mediated expression of excision repair cross-complementing gene 1 (ERCC1) [86]. ROS-mediated downregulation of miR-27a, miR-17-5p, and miR-20a was observed upon treatment with curcumin. These miRNAs target proteins such as ZBTB10 and ZBTB4, characterized by the presence of zinc finger and BTB domain. These proteins are found to suppress Sp proteins Sp4, Sp1, and Sp3, which play crucial roles in the development and advancement of colon cancer [87]. Increased expression of miR-130a has been linked to chemotherapy resistance and poor clinical outcomes in colon cancer patients. Recent studies report that curcumin downregulates miR-130a, resulting in the activation of the Wnt/β-catenin pathway in colon cancer. Additionally, curcumin induces elevated expression of miR-34a and suppresses expression of the oncogenic miR-27a, modulating downstream targets and leading to cell cycle arrest and apoptosis in CRC cells [88]. Curcumin affects CRC by downregulating several miRNAs, including miR-21, miR-20a, miR-27a, and miR-17, while upregulating miR-34. These miRNAs are essential regulators of cellular functions, including growth, survival, proliferation, resistance to chemotherapy, and migration [89,90]. Curcumin has been shown to modulate miRNA expression profiles in CRC cells, promoting tumor suppression. It upregulates tumor-suppressive miRNAs, such as miR-34a, while downregulating oncogenic miRNAs, including miR-27a and miR-130a. These changes contribute to reduced CRC cell proliferation, enhanced apoptosis, and increased sensitivity to chemotherapy, highlighting curcumin’s role in epigenetic regulation [88]. Another study on curcumin shows that it reduces lysine methylation levels in colon cancer cells by downregulating methyltransferases such as EZH2, MLL1, and G9a. This decline in lysine methylation is linked to impaired cell survival and the activation of apoptosis and ferroptosis, emphasizing curcumin’s potential in epigenetic regulation of CRC [91].

Concisely, curcumin, a polyphenol from turmeric, exhibits anticancer effects in CRC by modulating epigenetic and signaling pathways. It inhibits DNMT activity, suppresses oncogenic miRNAs (miR-27a, miR-130a), and upregulates tumor-suppressive miR-34a, leading to apoptosis and reduced proliferation. Curcumin also enhances chemotherapy sensitivity by regulating miRNAs like miR-137 and miR-409-3p. Additionally, it downregulates methyltransferases (EZH2, MLL1, G9a), reducing lysine methylation and promoting apoptosis and ferroptosis. These effects highlight curcumin’s role in epigenetic regulation and its therapeutic potential in CRC.

### Quercetin

Quercetin, a derivative of natural flavonoid glycosides, is commonly found in many fruits and vegetables. It is abundant in fruits and vegetables like apples, grapes, green tea, onions, etc [78,92]. Numerous health advantages have been associated with it, such as anti-inflammatory, anti-hypertensive, anti-cancer, antioxidant, and neuroprotective properties. Recent research shows quercetin may influence epigenetic networks and alter canonical biochemical signaling pathways [93]. Quercetin as an epigenetic modulator includes inhibition of DNA methylation by demethylation of gene promoter reduction of HAT activity, reduced expression of DNMT1, and inhibition of HDAC [94]. Studies have shown that quercetin can restore the tumor-suppressing function of the p16^INK4a^ gene, a key cell cycle regulator that helps control cell proliferation. In cancer cells, p16^INK4a^ is silenced through DNA methylation, which involves adding a methyl group to the gene’s promoter region and inactivating its function. Quercetin has been demonstrated to demethylate this promoter region, reactivating the p16^INK4a^ gene and enabling it to inhibit cancer cell growth [81,95]. Quercetin has shown the ability to inhibit around 40% of growth in RKO human colon cancer cells by promoting the demethylation of the CDKN2A gene promoter, leading to increased gene expression at a concentration of 80 µM [80]. Quercetin exhibits its anti-cancer characteristic by suppressing the functional activity of NF-κB in Caco-2 and SW620 cells [96]. Quercetin has been shown to promote H3 acetylation in certain cancers by activating histone acetyltransferase (HAT) enzymes. Studies indicate that quercetin increases H3 and H4 acetylation on the promoters of genes involved in apoptotic signaling pathways [95]. Quercetin inhibits NF-κB acetylation by targeting the p300 hAT enzyme. Additionally, it promotes p^16INK4a^ activation through promoter demethylation, thereby suppressing colorectal cancer progression [97]. Quercetin decreases cell viability and induces apoptosis in HCT116 and HT-29 colon cancer cells by increasing the expression of negative regulators in proliferation pathways, including Sprouty2, PTEN, and SFRP1, which are direct targets of miR-27a, a microRNA whose high levels are suppressed by quercetin. Additionally, quercetin reduces the expression of miR-23a, miR-24-2, and their primary miRNA transcript, suggesting it modulates transcription across this entire gene cluster, potentially regulated by Sp1 [98].

In summary, quercetin, a natural flavonoid found in fruits and vegetables, exhibits various regulatory functions including anti-inflammatory, antioxidant, and anticancer properties. It functions as an epigenetic modulator by inhibiting DNA methylation, reducing DNMT1 expression, and suppressing HDAC activity. Quercetin restores tumor-suppressing functions by demethylating the p16^INK4a^ promoter, reactivating its role in controlling cell proliferation. It inhibits colorectal cancer cell growth by promoting *CDKN2A* gene demethylation, suppressing NF-κB activity, and enhancing histone acetylation. Additionally, quercetin modulates miRNA expression, downregulating oncogenic miRNAs like miR-27a, miR-23a, and miR-24-2, thereby inhibiting cancer cell viability and promoting apoptosis.

### Sulforaphane

Sulforaphane (SFN) is a phytochemical derivative of isothiocyanate found in cruciferous plants and demonstrates anti-carcinogenic, anti-inflammatory, and antioxidant properties and preventive effects on CRC. They occur in the form of glucoraphanin, which is a subtype of phytochemical known as glucosinolate [99,100]. SFN inhibits the cell cycle in various tumor cells by inhibiting HDAC activity, essential for triggering apoptosis [101]. Similarly, prior studies have highlighted that SFN is one of the most potent natural HDAC inhibitors. Such natural compounds can both induce apoptosis and shield DNA from oxidative damage. Trichostatin, a metabolite extracted from a fungus, is a well-known HDAC inhibitor utilized as a positive control in SFN investigations [102]. SFN and its metabolites were found to have chemo-preventive properties. The metabolites include SFN-cysteine and SFN-N-acetylcysteine, a vital part of the mercapturic acid pathway in cells [103]. Increased Nrf2 expression in Caco-2 cells may be caused by SFN’s suppression of DNMT, which demethylates CpG sites in the Nrf2 promoter region and increases Nrf2 expression. Research suggests that the DNMT inhibitor 5-aza, combined with the HDAC inhibitor TSA can reverse epigenetic modifications and enhance the expression of Nrf2 and its downstream enzymes, which are associated with antioxidative and detoxifying properties [104]. Additionally, the motility and migration of CRC cells are hindered by the application of SFN. At the molecular level, SFN led to upregulation of erythroid 2 like 2 (Nrf2), nuclear factor and UDP glucuronosyltransferase 1A (UGT1A) expression. SFN exhibits chemopreventive properties *via* modulating anti-proliferative pathways and Nrf2-mediated detoxification in CRC [105]. Studies have reported that SFN when used at concentrations of 3–15 μM, can reduce HDAC activity in the HCT116 cell line. Similarly, other studies have shown that SFN inhibits HDAC activity and increases the expression of proteins involved in apoptosis, including p21 and Bax [106]. Nrf2 is activated by interacting with Keap1 and epigenetic mechanisms when SFN is used. SFN activated Nrf2 promoter epigenetically by inhibiting HDACs (1,4,5,7) and DNMTs (1 and 3a), which further decreases the methylation level of CpGs and increases histone 3 acetylation at Nrf2 promoter. K4 of histone H3 is tri-methylated by mixed lineage leukemia (MLL) protein, leading to transcriptional activation, and MLL knockdown leads to the downregulation of Nrf2 and HO-1 in Colon cancer cells, further reinforcing the epigenetic regulation of Nrf2 [107]. A cysteine metabolite of SFN was first described about 20 years ago, highlighting its HDAC activity inhibition property in *in vitro* experiments. This epigenetic effect was also observed in an *in vivo* study on an *Apc*^*Min/+*^ mouse model with intestinal polyps and other tissues. In a small human trial, participants’ consumption of fresh broccoli sprouts led to a rapid and temporary reduction in HDAC activity, accompanied by histone hyperacetylation in peripheral blood mononuclear cells. SFN exhibits epigenetic regulation in various cancers by inducing DNA methylation changes of cell cycle regulators such as p21 and cyclin D2, TSGs, namely *RARβ2, CDH1, DAPK1, PTEN*, and *GSTP1*, and pro-apoptotic BAX, and further decreases expression levels of DNMTs leading to cell cycle arrest and apoptosis. Non-coding mRNAs are also affected by the SFN and other isothiocyanate (ITCs), thereby inhibiting cell migration, cell proliferation, invasiveness, and epithelial-to-mesenchymal transition (EMT) [108]. A study revealed that SFN treatment reduced cell density, markedly inhibited cell viability, and triggered apoptosis in CRC cells. Moreover, SFN significantly downregulated oncogenic miR-21, HDAC, and human telomerase reverse transcriptase (hTERT) at the mRNA, protein, and enzymatic levels in CRC cells [109].

Briefly, SFN, a phytochemical from cruciferous vegetables, exhibits anti-cancer, anti-inflammatory, and antioxidant properties, particularly in CRC. It is a potent natural HDAC inhibitor, promoting apoptosis and protecting DNA from oxidative damage. SFN modulates epigenetic mechanisms by inhibiting DNMTs and HDACs, leading to increased expression of tumor suppressor genes (e.g., *p21, PTEN* etc) and Nrf2, a key regulator of detoxification pathways. It also reduces CRC cell viability, inhibits migration, and suppresses oncogenic miRNAs like miR-21. Both *in vitro* and *in vivo* studies confirm SFN’s ability to regulate the cell cycle and induce apoptosis through epigenetic modifications.

### Resveratrol

Resveratrol (RVT, 3,5,4°C-trihydroxystilbene) is a natural polyphenol. This natural compound can be found in grapes, plums, apples, peanuts, and blueberries and is considered one of the most extensively investigated food components. It reduces DNMT expression by silencing the TSG. Additionally, resveratrol-salicylate, a synthetic derivative, could significantly inhibit DNMT. Resveratrol analogs have been shown to selectively inhibit DNMT1, DNMT3a, and DNMT3b [110]. Inhibition of HDACs with induction of regulatory T cells (Treg) was exhibited by sodium butyrate and resveratrol. Analysis of datasets from The Cancer Genome Atlas (TCGA) showed that elevated expression of Treg-specific transcription factors FoxP3 or anti-inflammatory cytokine interleukin 10 (IL-10) leads to increased survival in patients with CRC. These data suggested an anti-inflammatory T-cell response in the gut microbe, leading to the reduction of inflammation-driven CRC [111]. Recent studies have demonstrated that resveratrol upregulated miR-34a, a homolog of miR-34c, leads to the suppressed development of human CRC cells. Studies have shown that the anti-CRC property exhibited by resveratrol is augmented in the presence of p53 in both *in vitro* and *in vivo* conditions by activating miR-34c-KITLG. It has also been observed that resveratrol-induced upregulation of miR-34c enhances chemosensitivity to oxaliplatin treatment in CRC. Resveratrol exhibits inhibition of cell growth, invasion and EMT-related gene expression by elevating the expression of miR-200 in HCT-116 CRC cells [112]. Several studies demonstrated that resveratrol is a powerful antioxidant and exhibits anti-cancer properties [113]. *In vivo*, studies on mice injected with resveratrol demonstrated an increased level of miR-96 that targets the *KRAS* oncogene. Resveratrol regulates miRNA-96 expression, activating the MAPK/ERK1/2 signaling pathway and inhibiting growth and apoptosis in colon cancer DLD-1 cells [114]. A study showed that resveratrol induces apoptosis in CRC cells by modulating the interaction between Sirtuin 1 (Sirt-1), a histone deacetylase, and the tumor suppressor p53. Resveratrol treatment inhibited Sirt-1 expression and activity, leading to increased p53 acetylation and activation. This, in turn, upregulated pro-apoptotic gene expression, triggering apoptosis in CRC cells. Notably, these effects were observed only in p53-proficient CRC cells, highlighting p53’s critical role in resveratrol’s mechanism of action [115].

Concisely, resveratrol, a natural polyphenol found in various fruits and nuts, exhibits significant anti-cancer and epigenetic regulatory properties. It suppresses DNMT expression and selectively inhibits DNMT1, DNMT3a, and DNMT3b, while its synthetic derivative, resveratrol-salicylate, enhances this inhibition. Additionally, resveratrol modulates HDAC activity and promotes anti-inflammatory T-cell responses, potentially reducing inflammation-driven CRC. It upregulates miR-34a and miR-34c, suppressing CRC progression and enhancing chemosensitivity to oxaliplatin. Moreover, resveratrol influences miR-96 to target oncogenic KRAS and modulates the Sirt-1/p53 pathway, leading to apoptosis in p53-proficient CRC cells. These findings highlight its potential as a chemopreventive and therapeutic agent.

### Epigallocatechin-3-gallate (EGCG)

A naturally occurring polyphenol called epigallocatechin gallate (EGCG) is extracted from green tea leaves. Its anti-tumor properties have drawn a lot of interest lately. Research studies have demonstrated that EGCG can suppress tumor growth, invasion, migration, and progression by inducing apoptosis [116,117]. Numerous studies highlight EGCG’s role in regulating the cancer cell cycle, growth, and apoptosis by modulating various signaling pathways. However, in this review, we specifically focus on its epigenetic regulatory functions in CRC, including alterations in DNA methylation, histone modifications, and non-coding RNA (microRNA) expression, which contribute to its anticancer effects [117]. Previous studies suggest that EGCG reactivated methylation-silenced genes in cancer cells like *KYSE150, PC3*, and HT-29 by inhibiting DNMT activity, showing anti-cancer properties by reversing the silenced genes. The probable mechanism of EGCG as a DNMT inhibitor might be due to the gallic acid portion of the D ring in the polyphenol EGCG exhibiting interaction with the cytosine active site on the DNMT enzyme. Evidence from prior literature suggests that sodium butyrate and EGCG combined application exert cell apoptosis and induce cell cycle arrest in CRC [118]. According to current studies, it has been noted that EGCG holds the potential to suppress the Wnt/β-catenin pathway in CRC. The chemoprotective activity was also exhibited by EGCG, possibly by PP2A and GSK-3β independent mechanisms in colon cancer cells [119]. Data from previous studies supports that EGCG inhibits the gene-silencing potential of HDACs and DNMTs. Butyrate also inhibits HDACs and induces colon cancer cell differentiation. ECGC demonstrated the capability to inhibit butyrate-induced differentiation of HT-29 cells [120]. It has been observed that heightened methylation of both active and repressive histones H3K4me3 and H3K9me3, respectively, and also increased histone acetylation of H3K9/14ac and H3ac upon treatment with EGCG. It has also been highlighted that catechin acts as an HDAC inhibitor in both cellular and cell-free models. Reduction of the expression of heterochromatin binding proteins such as HP1α and HP1γ was found to affect chromatin architecture due to EGCG application. Further, EGCG demonstrated chromatin relaxation and exerts the potential to regulate epigenome modulators such as HDAC5, HDAC7, CREBP, p300, LSD1 or KMT2A [121]. Furthermore, EGCG exerts antitumor effects in colon cancer by modulating epigenetic regulators such as DNMTs, HDACs, and HATs. In HCT-116 and HT-29 cells, EGCG treatment induced S-phase cell cycle arrest and apoptosis, along with upregulated DNMT3b and downregulated HDAC3 expression. These alterations suggest that EGCG helps restore global and gene-specific methylation and acetylation patterns, supporting its chemopreventive potential in colorectal cancer [122].

In summary, EGCG, a polyphenol from green tea, exhibits strong anticancer properties by modulating epigenetic mechanisms in CRC. It inhibits DNMT activity, leading to the reactivation of silenced tumor suppressor genes, and suppresses the Wnt/β-catenin pathway. EGCG also acts as an HDAC inhibitor, affecting chromatin architecture by reducing heterochromatin-binding proteins and altering histone modifications. Additionally, it induces apoptosis and S-phase cell cycle arrest in CRC cells while restoring global methylation and acetylation patterns. These findings highlight EGCG’s potential as a chemopreventive agent in CRC.

### Anthocyanins

Anthocyanins are flavonoid compounds that impart color to fruits and vegetables and also exhibit anti-cancer properties, and are used as an appetite stimulant. They are abundant in berries, particularly strawberries and black raspberries (BRB) [123]. The BRB anthocyanins influenced acetylation levels by decreasing Sirtuin 1 (*SIRT1*) expression while increasing the expression of *MOF* and *EP300*. This led to an increased acetylation of lysine residues on histone H4 (H4K5, H4K12, and H4K16). Additionally, treatment with BRB anthocyanins significantly up-regulated the expression of ac-p65 and activated the NF-κB signaling pathway. This activation subsequently increased *BAX* expression while reducing the expression of Bcl-2, cyclin-D1, c-myc, and NLRP3, promoting CRC cell cycle arrest, inducing apoptosis, and alleviating inflammation [124]. Study on cell lines including HCT116, HT29/219, SW480 and LS180, when subjected to treatment with anthocyanin at IC20 and IC50 concentrations, demonstrated a significant downregulation in transcript levels of DNMT1 and DNMT3B in a dose-dependent manner. Treatment with blackberry extract also induced demethylation of the promoter region of *SFRP2* and *P16* genes in CRC cell lines. However, it is also being observed that in cell lines viz. HT29/219, SW742 and SW480, berry extract upregulated Dnmt3a expression [125]. A study on an AOM/DSS-induced mice model showed downregulated expression of miR-483-3p upon treatment with BRB anthocyanins. Further data from PCR and Western blot analysis post-treatment with the extract showed that there was an upregulated expression of Dickkopf3 (DKK3), a potential target of miR-483-3p as predicted by *in silico* study when miR-483-3p was downregulated. BRB showed anthocyanin-mediated anti-CRC properties by downregulating downstream factors of the DKK3 signaling pathway, including Wnt/β catenin, and was found to regulate miR-483-3p. Thus, this supports the idea that miR-483-3p could be a potential target of CRC [126]. Another study demonstrated that these BRB anthocyanins upregulated the expression of miR-24-1-5p, which was suggested to negatively regulate the Wnt/β-catenin protein levels in the cells [127]. Similarly, another study demonstrated that black raspberry anthocyanins suppress human colon cancer cell growth by upregulating miR-24-1-5p. This, in turn, downregulates β-catenin, a crucial regulator of the Wnt signaling pathway, indicating that anthocyanins may exert their anticancer effects through miRNA-mediated epigenetic regulation [128]. A 2011 phase I pilot investigation revealed that oral administration of BRB resulted in reduced methylation of TSGs, specifically Wnt inhibitory factor (*WIF1*) and *SFRP2*. Both genetic and epigenetic biomarkers are modulated by BRBs in tissue from CRC patients. BRBs also modulate the expression of genes involved in the Wnt pathway like β-catenin, E-cadherin and TSGs like *SFRP2* and *WIF1 by* demethylating. Inhibition of DNMT1 was likely due to demethylation of genes, although methyltransferase enzymes might have also been affected. BRBs, when used to treat patients with rectal adenocarcinomas for a short period of about 2 weeks, showed reduced response. Suggesting BRB effectiveness could be enhanced by increasing the treatment period or developing a localized delivery system targeting rectal tissues [129]. Anthocyanins produced from BRB were found to inhibit the activity of DNMT1 and DNMT3B in certain human colon cancer cell lines. Additionally, these anthocyanins were discovered to demethylate the promoter regions of TSGs, namely *CDKN2A, SFRP5, SFRP2* and *WIF1*. As a result, the β-catenin expression levels were significantly decreased [130].

Briefly, anthocyanins, flavonoid compounds found in berries, exhibit anti-cancer properties by modulating epigenetic mechanisms in CRC. The BRB anthocyanins regulate histone acetylation by decreasing *SIRT1* and increasing *MOF* and *EP300*, leading to apoptosis and reduced inflammation. They also downregulate DNMT1 and DNMT3B, promoting the demethylation of TSGs like *SFRP2* and *P16*. BRB anthocyanins influence miRNA expression, notably suppressing miR-483-3p and upregulating miR-24-1-5p, which inhibits Wnt/β-catenin signaling. Clinical studies suggest their potential for CRC treatment, though prolonged or targeted administration may enhance efficacy.

## Natural compounds modulating the epigenetic regulation of inflammasome and autophagy in CRC

Inflammasomes exhibit a dual role in CRC, functioning as both tumor promoters and suppressors depending on the tumor microenvironment. While inflammasome activation can enhance anti-tumor immunity by inducing pyroptosis and recruiting immune cells, chronic inflammasome signaling contributes to a pro-tumorigenic microenvironment through sustained inflammation and the release of IL-1β and IL-18, which may drive epigenetic modifications associated with CRC progression [131]. A study on CRC cell lines (RKO, HT29, and HCT116) revealed that curcumin selectively alters DNA methylation in a subset of partially methylated genes in a time-dependent manner. In contrast, 5-aza-CdR induces non-specific global hypomethylation. These findings highlight curcumin’s potential chemopreventive role in CRC through targeted epigenetic modulation [132]. Similarly, another study on CRC cell lines, including HCT116, SW480, LoVo, and HT29, demonstrated that curcumin treatment significantly reduced cell viability while increasing the SubG1 phase across all cell lines, indicating apoptosis activation. Regarding pyroptosis, NLRP3 inflammasome components were upregulated in HCT116 and, to a lesser extent, in SW480 cells, whereas no activation was observed in HT29 and LoVo cells. These findings suggest that while curcumin effectively induces apoptosis, its impact on NLRP3 inflammasome-mediated pyroptosis varies among CRC cell lines [21,133]. A study using the azoxymethane-dextran sulfate sodium (AOM-DSS) induced mouse model demonstrated that DNA methylation levels in a group of inflammatory genes were reduced in the AOM-DSS group but restored upon curcumin treatment, was validated by pyrosequencing. Among the differentially expressed genes, *Tnf* was particularly noteworthy, exhibiting reduced DNA CpG methylation in the AOM-DSS group. Notably, curcumin treatment reversed this AOM-DSS-induced demethylation of *Tnf*. These methylation changes correlated with alterations in *Tnf* expression observed in RNA sequencing. This study highlights the epigenomic modifications in DNA CpG methylation associated with CAC inflammation and curcumin’s potential to reverse these epigenetic alterations. Further clinical studies are warranted to explore curcumin’s epigenetic effects in this context [134].

Quercetin induces CRC cell death through gasdermin D (GSDMD)-mediated pyroptosis. The study demonstrated that quercetin treatment upregulated NIMA-related kinase 7 (NEK7), facilitating NLRP3 inflammasome assembly and GSDMD cleavage. Notably, NEK7 silencing promoted both *in vitro* and *in vivo colon cancer cell growth*, highlighting its critical role in quercetin-induced pyroptosis [135]. Arctigenin, a bioactive compound derived from *Fructus arctii*, inhibits cell proliferation across various cancer types, potentially by inducing autophagy, while also alleviating colitis-associated symptoms. Metabolomic analysis revealed that arctigenin suppresses NLRP3 inflammasome activation and downregulates fatty acid oxidation (FAO) in macrophages [21]. Despite these promising findings, the precise mechanisms underlying the epigenetic regulation of inflammasomes and autophagy in CRC by natural compounds remain largely unexplored. Current research lacks comprehensive studies on this aspect, highlighting the need for further investigations to elucidate the molecular mechanisms linking natural compounds, autophagy, and epigenetic alterations in CRC.

## Bioavailability of natural compounds possibly affect their efficacy

The bioavailability of a compound refers to the amount or dose of the substance which has an active effect when induced into the circulation. Research trials have shown that many natural compounds like curcumin are safe even at high doses, but exhibit poor bioavailability [136]. The primary reasons for the low bioavailability of natural compounds in tissues include their rapid elimination from the body, high rate of metabolism, low intrinsic activity, poor absorption, and inactivity of metabolic products [137]. Natural compounds like genistein bioavailability can be enhanced using a solid-liquid particulate drug delivery system to improve stability, solubility, and controlled drug release from the formulation. Another approach could be hydrocolloids, in which poor-soluble drugs are entrapped in lipophilic carriers like polyethylene glycol (PEG), carrageenan, and polyvinyl pyrrolidone (PVP) is known to improve solubility, pharmacokinetics, and bioavailability [75]. Previous studies have reported that quercetin has poor bioavailability. However, recent studies have reported that this low bioavailability can be enhanced by methods like structural modification, including complex ionic complex formulation, glucoside-sulfate conjugates, quercetin-germanium nanoparticles, glucan-quercetin conjugate, and calcium phosphate-quercetin nanocomposite (CPQN), thereby enhancing its antioxidant activity compared to free quercetin form [138]. Studies have reported that SFN is highly unstable and rapidly metabolizes when exposed to oxidants, heat, and light. Moreover, it has also been noted that it gets degraded even during long-term refrigeration, albeit slowly compared to room temperature. Therefore, various studies focused on overcoming this challenge have reported that SFN could be stabilized with PEG, microencapsulation embedding, and cyclodextrin inclusion complex approaches [139]. Previous studies have reported that anthocyanin has shown poor bioavailability. A comparative study on two different formulations of anthocyanin illustrated that pelargonidin-3-O-rutinoside (PgR) showed enhanced bioavailability than pelargonidin-3-O-glucoside Pg3G [140]. Using natural compounds in clinical studies remains limited due to their associated bioavailability challenges. Despite the encouraging outcomes of current studies in this field on enhancing bioavailability in both *in vitro* and *in vivo* investigations, additional investigation is required to determine and establish their potential therapeutic use. Some natural compounds targeting epigenetic modulations in CRC are discussed here and listed in Table 2.

**Table 2.** Natural compounds as epigenetic modulators and their mechanism of action.

Name of the compound	Origin	Target	Mechanism	Ref
Genistein	A major bioactive compound in soy products	Target the Wnt/β-catenin signaling pathway, histone modification, DNA methylation	Reduced methylation at CpG sites of Wnt5a and sFRP genes. Increased acetylation in the promoter site of DKK1 gene by histone H3. Demthylate WIF1	[82,141]
Curcumin	Polyphenolic derivative product of turmeric	Regulates NF-κB, Jak/STAT signaling pathway	Downregulates MDM2, stimulates p53 and H3. Inhibits HDAC1. Regulates miR-21, miR-27, miR-34 etc.	[88,89]
Quercetin	Flavonoid obtained from fruits and vegetables such as apples, onions, red grapes, green tea leaves	PI3K-AKT, AKT/mTOR, RAS, Wnt/β-catenin signaling pathways	Upregulates miR-27a. Activates HATs. Inhibits the activity of DNMTs, HDACs, and HMTs.	[98,142,143]
Sulforaphane	Bioactive compound found in cruciferous vegetables	Histone modification and DNA methylation	Promotes the enzymatic activity of glutathione S-transferases (GSTs), UDP-glucuronosyl transferases (UGTs), NAD(P)H: quinone oxidoreductase 1 (NQO1). Decreases *DNMT1* expression, DNA methylation and histone modification	[105,144]
Resveratrol	Polymer from the family of viniferins	Class III HDAC and DNMT inhibitor	Evelates Sirtuin 1 (SIRT1) expression	[145]
Epigallocatechin-3-gallate (EGCG)	Polyphenol found in green tea leaves	Histone modification and methylation	Reduces the expression of DNMT3A and HDAC3. Restores RXRα expression	[146,147]
Anthocyanins	Richly found in berries especially strawberries and black raspberries	Targets the WNT signalling pathway	Suppresses the activity of DNMT1 and DNMT3B. Demethylates promoter region of tumor suppressor genes, *CDKN2A, SFRP5, SFRP2, WIF1 and β-catenin*	[125,148]

## Synergistic actions of natural compounds with main stream CRC drugs

Conventional treatment regimens such as chemotherapy and radiotherapy are associated with low cure rates and serious side effects. Numerous *in vitro* and *in vivo* studies have shown that phytochemicals derived from fruits and vegetables possess potent anticancer, anti-inflammatory, and antioxidant properties. By selectively targeting cancer-related molecular pathways, these compounds prevent CRC from initiation and progression [149]. Additionally, prior studies have demonstrated that polyphenols can modulate different molecular mechanisms to improve the effectiveness of therapeutic medications and reduce chemo-resistance [150]. Despite the number of advantages of 5-Flurouracil (5-FU), clinical use remains restricted due to the emergence of drug resistance. A study conducted on the HCT-8 colorectal cell line demonstrated that curcumin increases the sensitivity of 5-FU for the colorectal cell line by downregulating the expression of P-GP and HSP-27, both of which are associated with multidrug-resistant (MDR) proteins [151]. Another study demonstrated that curcumin can enhance the anticancer efficacy of 5-FU by suppressing mdr1 gene expression. In 5-FU-resistant CRC cell lines, curcumin inhibits cell proliferation and induces apoptosis by downregulating EMT and up-regulating miRNAs that suppress EMT [152].

In contrast to treatment with either CIS or CUR alone, a prior study showed that curcumin and CIS effectively synergize to decrease the proliferation of cisplatin-resistant HT-29 colon cells, with more favorable effects reported [153]. A study involving azoxymethane (AOM)-induced CRC mice, as well as Caco2 and SW480 cell lines, demonstrated that the combined treatment of chemotherapy drugs such as 5-fluorouracil (5-FU) or celecoxib (Cel) at varying concentrations, alongside the natural compound anthocyanin, effectively inhibited cell proliferation and reduced tumor numbers in AOM-induced CRC mice. Mechanistic analysis revealed an upregulation of PTEN, downregulation of Enhancer of Zeste Homolog 2 (EZH2), and inhibition of the AKT signaling pathway [152,154]. While demonstrating no toxicity toward CCD-180Co normal colon cells, a study examining the effects of blueberry extract (BE) in combination with oxaliplatin (OX) on HCT-116 cells showed considerable inhibitory action. According to flow cytometry analysis, this combination of treatments caused HCT-116 cells to undergo G0/G1 cell cycle arrest, elevate reactive oxygen species (ROS) levels, encourage apoptosis, and lose their mitochondrial membrane potential. AKT/BAD/BCL-2 signaling pathway modulation, downregulation of cyclin D1 and CDK4, and activation of caspases 3 and 9 were further outcomes of the combination therapy [155]. A study showed that 5-FU and quercetin, when combined, exhibited a dramatic reduction in the proliferation of COLO 320DM CRC cells.

Furthermore, studies have shown that quercetin and cisplatin synergistically inhibited cell proliferation and triggered apoptosis by triggering the NF-κB signaling pathway [156]. In DLD-1 and HT-29 CRC cells, EGCG, in combination with either CIS or OX, efficiently suppressed proliferation and caused cell death. When combined with CIS or OX, EGCG had a synergistic effect that induced autophagy. This was evidenced by the accumulation of LC3-II protein, the rise in acidic vesicular organelles (AVOs), and the formation of autophagosomes [157]. 5-FU-resistant HCT116 cells were demonstrated to undergo apoptosis when resveratrol and the chemotherapeutic drug 1,3-Bis (2-chloroethyl)-1-nitrosourea were combined.

Additionally, resveratrol sulfate enhanced the effectiveness of oxaliplatin. However, conflicting findings suggest that resveratrol and OX may not necessarily result in chemosensitization. Consequently, further research is required to validate the possibility of resveratrol in enhancing the susceptibility of CRC cells to chemotherapy [158]. Genistein was given, in addition to the treatment FOLFOX, to 13 participants in a clinical trial. Mild adverse effects such as nausea, headaches, and hot flashes were recorded, with one incidence of grade 3 hypertension. Interestingly, genistein did not enhance adverse effects associated with chemotherapy, indicating that it is well tolerated in combination therapy [71,83]. SFN, both alone and in combination with FOLFOX, significantly reduced the viability of highly metastatic human colon cancer cells CX-1, induced apoptosis, inhibited spheroid formation, and suppressed ALDH1 activity. However, SFN also increased mrp2 expression and protein levels. Overall, the combination of SFN with FOLFOX demonstrated additive anticancer effects against highly metastatic human CRC cells *in vitro* [159].

## Challenges associated with the use of natural compounds

The major challenges associated with natural compounds are discussed in the following subsections. Firstly, the food intake. Once after the diagnosis of cancer, patients tend to change their dietary intake after consulting their oncologists. Though there are numerous plant products identified to enhance and support cancer therapy, so far, the FDA has not stated any dietary supplement to prevent cancer. This becomes a big challenge for the oncologists to give their patients proper advice on their diet plans and food intake [160]. When a natural product or its bioactive compound is discovered to have anti-cancer properties, it has to undergo various preclinical trials. These involve complex tests in humans and also post-trial regulatory approval by the FDA. These processes are highly expensive and time-consuming, which need millions of dollars, and it takes a minimum of 10–15 years for a single product to be approved. It is estimated that only 12% of the compounds can get the final approval [161]. Several other challenges involve harvesting and culturing such natural compounds in an artificial environment, such as a lab for their production. The results might be manipulated if the contamination rates are not adequately maintained. Such errors can cause the product with potential advantages to get rejected or disapproved by the FDA. So, maintaining sterility and an optimum work environment is necessary for error-free results. The next challenge is the availability of active compounds in extracts. Natural compounds are composed of many other admixtures. From a large scale of culture, very low amount of a selective product is obtained from the crude. The isolation and purification of such a small concentration of active compound becomes yet another level of trial. It requires adequate procedures and ideal equipment. The calibration of such equipment and instruments must be under watch and regularly maintained. After the purification of crude, the products might waste out toxic compounds. The management of byproducts produced during extraction from the crude extracts is necessary, and proper disposal of those compounds might be challenging. Bio-waste management guidelines vary from compound to compound; in such cases, good knowledge is required before handling natural compounds or carrying out procedures. Understanding the bioavailability of these compounds also is a demanding task as the absorption of such a product inside our body is as important as determining the product’s dosage to be consumed as part of the treatment.

## Discussion

Epigenetics is one of the radical factors influencing CRC progression. In recent times, abundant research has been undertaken to explore the epigenetic mechanisms of action that lead to CRC. Several works have been published that report the potential of targeting epigenetic regulation in CRC treatment. Currently, many plant-based products that have properties that target these epigenetic pathways are being investigated and studied to understand better their specific targets, physicochemical properties, and mechanism of action. The scope of such research promises to overcome the limitations and harmful effects of commercial drugs. There are enormous advantages in using natural compounds in contrast to synthetic molecules currently employed as conventional therapy for cancer treatment. The natural compounds attribute a wide range of chemical compounds with good structural stability. Various research studies have discovered the anti-cancer properties of many such natural compounds, including the ability to regulate epigenetic modifications in CRC. Genistein, curcumin, quercetin, SFN, resveratrol, EGCG, anthocyanins, and many other dietary compounds and polyphenols targeting DNA methylation status, histone modifications, and microRNAs involved in CRC carcinogenesis have been reviewed in the paper. The use of natural compounds in the treatment of CRC, along with conventional therapy, has been gaining more attention due to the less toxicity and more efficacy associated with it.

But along with those advantages, there are a few challenges associated with it. The central dispute with these natural compounds is their source and availability. Manufacturing of such products on a large scale requires high quality control and their safety must be guaranteed. Though the natural compounds are, to an extent, harmless, their side effects when given in combination with combined therapy are still not very clear. In 2019, Phase I/II clinical trials were carried out to evaluate the efficacy and safety of introducing genistein, combined with FOLFOX (standard chemotherapy) with or without bevacizumab, for treating metastatic CRC. The study involved the treatment of 13 patients, and it was shown that combining genistein with chemotherapy potentially enhanced treatment efficacy compared to standard chemotherapy alone. The study conducted by Pintova et al., 2019 did not find any significant toxicity. However, using natural compounds in conjunction with conventional therapy in clinical trials is a recent phenomenon [83]. Consequently, the protocols for their application and the delivery of these items to patients are still being formulated. Yet another challenge in using natural compounds is the time lag in their action, which is comparatively slower than commercial drugs. These challenges might limit their utilization, but they will potentially be invaluable shortly due to emerging advancements in technology and research.

## Conclusion

Natural compounds with epigenetic regulatory properties offer promising avenues for CRC treatment. Bioactive molecules such as genistein, curcumin, quercetin, sulforaphane, resveratrol, EGCG, and anthocyanins influence DNA methylation, histone modifications, and non-coding RNAs, thereby modulating CRC progression. Many of these compounds have demonstrated efficacy in both *in vitro* and *in vivo* models. Notably, curcumin, genistein, anthocyanins, resveratrol [162,163], and EGCG [164,165] have been evaluated in clinical trials for various diseases. Among them, curcumin was investigated in a randomized, double-blind, placebo-controlled clinical trial involving 44 patients with non-alcoholic fatty liver disease, exhibiting epigenetic regulatory effects [166]. Genistein, in combination with FOLFOX, showed a synergistic effect in a Phase I/II pilot study with 13 patients with metastatic CRC, though its specific epigenetic role was not elucidated [83].

Additionally, a Phase I pilot study involving 20 patients reported that BRB/anthocyanin treatment for approximately four weeks led to the demethylation of tumor suppressor genes *SFRP2, SFRP5*, and *WIF1*, key Wnt pathway inhibitors, along with *PAX6a*, correlating with reduced DNMT1 expression. BRB/anthocyanins also modulated genes related to Wnt signaling, proliferation, apoptosis, and angiogenesis in a protective manner. While demethylation was not observed in all treated patients, secondary outcomes highlight the need for further investigation into BRBs for CRC prevention [129].

Despite these promising findings, further research and clinical validation are imperative to facilitate the integration of these compounds into mainstream CRC treatment. Future studies should focus on translating *in vitro* findings to clinical settings, optimizing dosage and treatment duration, improving bioavailability and establishing comprehensive safety profiles through well-designed clinical trials.

## Future Perspective

The field of cancer epigenetics is so vast. Integrating comprehensive epigenomic profiling with advanced bioinformatics tools can unveil novel epigenetic biomarkers for early diagnosis and personalized treatment strategies. The new biotechnological tools have offered next-level epigenetic biomarkers to detect cancer in the early stage. It has also helped to analyze and monitor cancer progression and predict its associated risk factors. Additionally, the exploration of natural compounds, such as polyphenols, flavonoids, and alkaloids, offers a rich reservoir of bioactive molecules capable of modulating epigenetic mechanisms, including DNA methylation, histone modification, and non-coding RNA regulation. Future research should focus on elucidating the precise molecular pathways through which these natural compounds exert their effects, optimizing their bioavailability and efficacy, and conducting rigorous clinical trials to establish their therapeutic potential. Approaches such as the addition of polar functional groups to the molecules have significantly increased the bioavailability of natural compounds with poor solubility. Other approaches like pH adjustment are also done on ionizable compounds to improve their tissue accessibility. Currently, adjuvants are being used, which interrupt the normal metabolism of natural compounds and help to provide longer circulation, and better permeability, thus increasing their bioavailability. The development and progress in nanomedicine have promised to substantiate high biocompatibility for natural compounds and their analogs in the near future.

Furthermore, the natural compounds, when combined with conventional chemotherapy drugs, have shown potential to enhance anticancer efficacy in CRC treatment. Compounds like curcumin and anthocyanin have been reported to synergize with drugs such as widely used CRC drugs such as 5-FU and OX. At the same time, genistein shows synergistic effect with FOLFOX in 13 patients enrolled with metastatic CRC. Improving drug sensitivity and inducing apoptosis. These combinations offer a promising complementary approach, though more clinical studies are needed to validate their effectiveness and optimize their use. By fostering interdisciplinary collaborations and leveraging cutting-edge technologies, we can pave the way for innovative, effective, and sustainable approaches to combat colon cancer through epigenetic modulation.

## Data Availability

The entire data set resulting from the studies analyzed for this study is depicted in the manuscript along with the figures.
